# Bridging the Gap in Eastern European Cystic Fibrosis Care: How Newborn Screening and Advanced CFTR Modulation Shape the Clinical Landscape in Western Romania

**DOI:** 10.3390/children13091178

**Published:** 2026-09-01

**Authors:** Cristian Phillip Marinău, Cristian Sava, Alin Iuhas, Ioana Mihaiela Ciucă, Liviu Laurențiu Pop, Larisa Niulaș, Ariana Szilagyi, Alexandru Jurca, Claudia Maria Jurca

**Affiliations:** 1 Department of Medical Disciplines, Faculty of Medicine and Pharmacy, University of Oradea, 410073 Oradea, Romania; 2 Pediatrics Department, Bihor County Clinical Emergency Hospital, 410167 Oradea, Romania; 3 Pediatric Department, University of Medicine and Pharmacy “Victor Babes”, 300041 Timișoara, Romania; 4 National Cystic Fibrosis Centre and Pediatric Pulmonology Ward, “Pius Brînzeu” County Emergency Clinical Hospital, 300226 Timișoara, Romania; 5 Department of Preclinical Disciplines, Faculty of Medicine and Pharmacy, University of Oradea, 410073 Oradea, Romania; 6 Regional Center of Medical Genetics Bihor, Bihor County Clinical Emergency Hospital (Part of ERN ITHACA), 410053 Oradea, Romania

**Keywords:** cystic fibrosis, newborn screening, CFTR modulators, elexacaftor/tezacaftor/ivacaftor, diagnostic lag, Eastern Europe, Romania, public health

## Abstract

**Highlights:**

**What are the main findings?**

• Newborn screening was associated with significantly earlier diagnosis than diagnosis prompted by symptoms.
• After national reimbursement, 76.1% of patients received triple-combination therapy, and lung function was generally preserved among those tested.

**What are the implications of the main findings?**

• At this center, treatment uptake after reimbursement approached levels reported in high-income Western European settings.
• Screening and treatment access should be complemented by sustained nutritional care, infection control, and national registry-based follow-up.

**Abstract:**

**Background/Objectives:** Cystic fibrosis (CF) care in Romania underwent a major transition in 2022 with the near-simultaneous introduction of newborn screening (NBS) and national reimbursement of CFTR modulator therapies. This study aimed to compare age at diagnosis between screen-detected and symptom-detected patients, describe clinical outcomes according to modulator status, and estimate regional case-detection ratios in Bihor County. **Methods:** This was a retrospective observational study with cross-sectional assessment of 46 CF patients followed at the National Cystic Fibrosis Centre in Timișoara during 2025. Nonparametric methods were used for exploratory comparisons. Case-to-live-birth ratios were estimated for pre-screening (2008–June 2022) and post-screening (July 2022–2024) periods in Bihor County. **Results:** Median age was 8.96 years (IQR 7.06–15.25); 58.7% of patients were male. Screen-detected patients (*n* = 5) were diagnosed earlier than symptom-detected patients (median 0.00 vs. 0.60 years; *p* = 0.016). F508del was present in 65.2% of alleles, with 22 distinct variants identified. Modulator therapy was received by 84.8% (76.1% elexacaftor/tezacaftor/ivacaftor). Median percent-predicted FEV_1_ (ppFEV_1_) was 99.0% (*n* = 38), comparable to ECFSPR 2024 pediatric values, while median BMI Z-score (−0.32) remained below Western European benchmarks. Chronic Pseudomonas aeruginosa and MRSA colonization rates were 11.4% (pediatric) and 15.2% (overall), respectively. ppFEV_1_ correlated strongly with BMI Z-score (*ρ* = 0.625, *p* < 0.001). The Bihor County case-to-live-birth ratio was 1:7820, with a descriptive 1.45-fold increase post-NBS. **Conclusions:** CF characterization in western Romania demonstrates rapid modulator uptake and preserved lung function while highlighting persistent gaps in nutritional status, microbiological burden, and case detection relative to Western European populations. Longitudinal nationally representative studies are needed to confirm these findings.

## 1. Introduction

Cystic fibrosis (CF) is one of the most common life-limiting autosomal recessive disorders among populations of European descent, with a birth incidence estimated at approximately 1 in 3000–6000 live births [1,2]. It is caused by pathogenic variants in the cystic fibrosis transmembrane conductance regulator (*CFTR*) gene. CFTR dysfunction disrupts epithelial ion and fluid transport, leading to dehydrated, viscous secretions in the respiratory, pancreatic, hepatobiliary, and gastrointestinal tracts and resulting primarily in progressive muco-obstructive lung disease and exocrine pancreatic insufficiency [3,4,5,6,7].

Over recent decades, newborn screening (NBS), nutritional and multidisciplinary care, and highly effective CFTR modulator therapy (HEMT) have transformed CF from a predominantly fatal childhood disease into a chronic multisystem disorder of adulthood [8,9,10]. A European Cystic Fibrosis Society Patient Registry (ECFSPR) analysis across 13 European countries estimated a median survival of 51.7 years (95% CI, 50.0–53.4), with a 46% lower mortality hazard in countries with the highest versus lowest healthcare spending [11]. Between 2011 and 2021, mean percent-predicted forced expiratory volume in 1 s (ppFEV_1_) increased from 85.0% to 94.2% in children and from 63.6% to 74.7% in adults, with additional improvement among people carrying the F508del variant following the introduction of elexacaftor/tezacaftor/ivacaftor (ETI) [12]. However, lower-income European countries showed no significant annual improvement in ppFEV_1_ or in the number of adults living with CF during the same period [12]. The 2024 ECFSPR Annual Data Report also demonstrates persistent country-level disparities in age at diagnosis and access to reimbursed CFTR modulator therapies [13,14].

In Romania, the public health infrastructure encounters significant structural impediments in the comprehensive management of this multisystem disease [9,15,16]. Before the implementation of universal NBS, CF diagnosis was predominantly symptom-driven, creating a risk of delayed recognition and advanced nutritional or pulmonary manifestations. While the Romanian healthcare system has achieved notable progress through national rare disease programs, geographic disparities in access to accredited centers of excellence and the socioeconomic burden of comprehensive, multidisciplinary care remain significant barriers [13].

Despite these persistent challenges, CF care in Romania entered a major transition in 2022. CF NBS was formally incorporated into the national public-health program in April 2022 and reportedly became operational in July 2022, while national therapeutic protocols for lumacaftor/ivacaftor (LI) and ETI were introduced in March and April 2022, respectively [17,18,19,20]. These parallel developments created a clinically relevant contrast between patients diagnosed symptomatically before universal screening—many of whom initiated modulator therapy after established disease—and a growing population of screen-detected infants with the potential to benefit from early monitoring and disease-modifying intervention.

Within this evolving context, a cross-sectional analysis complemented by retrospective registry data provides a valuable baseline characterization of the regional CF population during a period of substantial change in screening and treatment practices. The primary objective of this study was to describe the epidemiological profile, genetic diversity, and clinical characteristics of a CF cohort followed at a specialized center in Western Romania. Specifically, this study aimed to (1) compare age at diagnosis between screen-detected and symptom-detected patients; (2) describe nutritional status, respiratory function, hospitalization burden, and microbiological profiles according to current CFTR modulator status; and (3) estimate regional case-detection ratios in Bihor County across pre-screening and post-screening birth periods. These findings provide an initial regional perspective on the transition toward newborn screening and CFTR modulator therapy and may help contextualize persistent disparities relative to Western European CF populations.

## 2. Materials and Methods

### 2.1. Study Design and Population

**Figure 1:**
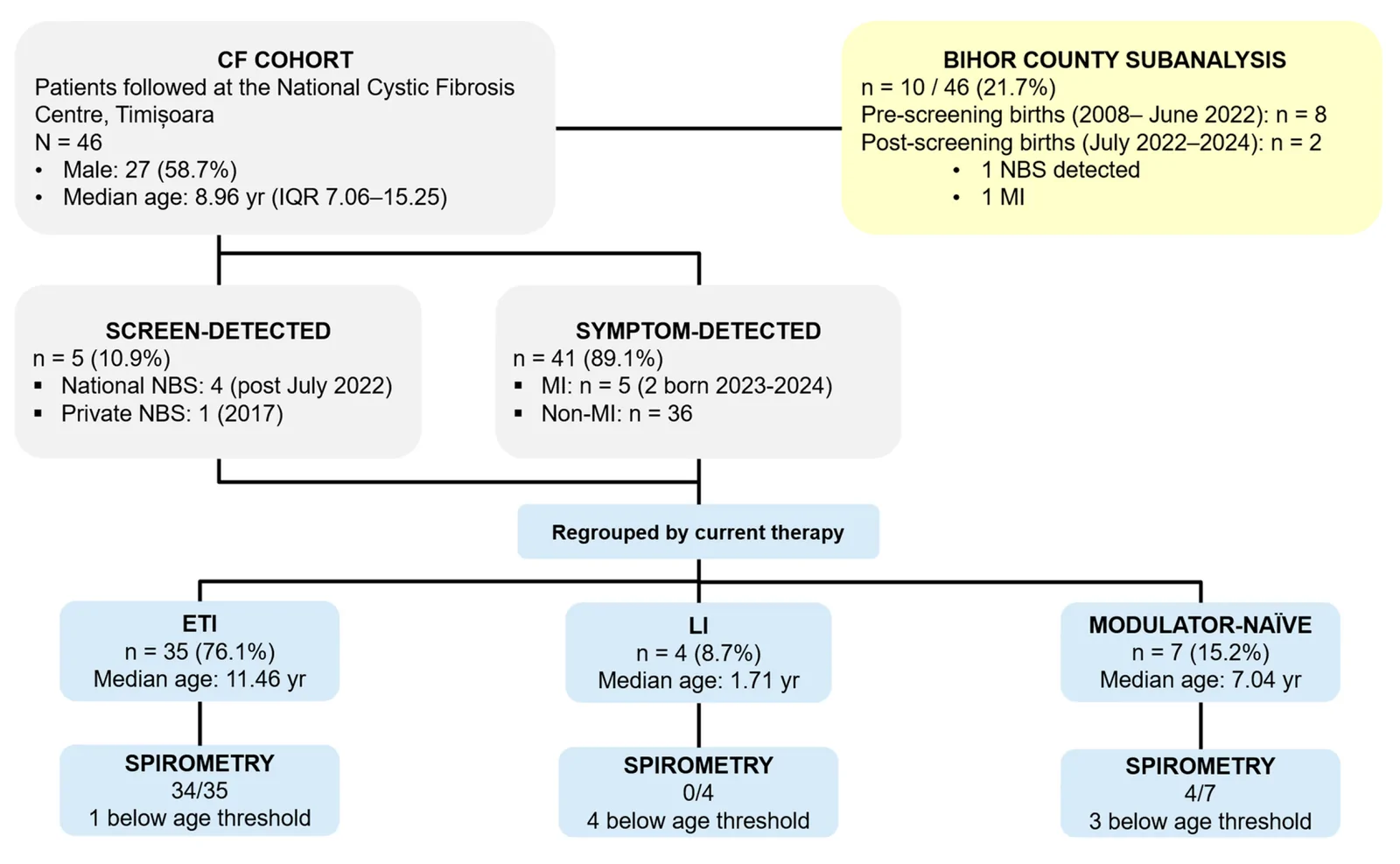
Flow diagram illustrating the derivation of analytic subgroups of the cohort. CF, cystic fibrosis; NBS, newborn screening; MI, meconium ileus; ETI, elexacaftor/tezacaftor/ivacaftor; LI, lumacaftor/ivacaftor; IQR, interquartile range; yr, years.

This retrospective observational study included a cross-sectional clinical and functional assessment of 46 patients followed at the National Cystic Fibrosis Centre in Timișoara during 2025, including 10 residents of Bihor County. A county-level epidemiological estimate was also conducted for Bihor using patients born during the 17-year period from 2008 to 2024. CF was confirmed based on clinical presentation, a sweat chloride concentration ≥ 60 mmol/L, and/or the identification of two disease-causing *CFTR* variants. All patients actively followed at the regional center were included, comprising 44 pediatric patients and two 19-year-olds who transitioned during longitudinal follow-up. The derivation of the analytic subgroups is presented in Figure 1.

### 2.2. Data Collection and Variables

Data were extracted from clinical observation charts and regional longitudinal monitoring registries. Variables included demographic and diagnostic characteristics, *CFTR* genotype, nutritional status, pulmonary function, microbiological findings, hospitalization burden, and current treatment.

Patients were classified as screen-detected when identified through national or privately funded NBS and as symptom-detected when diagnostic evaluation was initiated because of clinical manifestations. Births before July 2022 were assigned to the pre-screening period, whereas births from July 2022 onward were assigned to the post-screening period. Infants presenting with meconium ileus (MI) were classified as symptom-detected regardless of birth period because diagnostic evaluation was initiated clinically, generally before NBS results became available [20].

Current modulator treatment was classified as ETI, LI, or modulator-naïve. Treatment duration was estimated from available clinical records.

The best ppFEV_1_ value obtained during 2025 was recorded and calculated using the GLI-2012 multiethnic reference equations. Chronic respiratory infection with *Pseudomonas aeruginosa* (PsA), methicillin-sensitive *Staphylococcus aureus* (MSSA), or methicillin-resistant *Staphylococcus aureus* (MRSA) was defined according to ECFSPR criteria as isolation of the respective organism in >50% of respiratory samples collected during the preceding 12 months, with at least four samples required [21]. Hospitalization burden was measured as the cumulative number of inpatient days per patient during 2025.

### 2.3. Statistical Analyses

Statistical analyses were performed using JASP version 0.98.1 (JASP Team, Amsterdam, The Netherlands), Jamovi version 2.7.38 (The jamovi project 2026), and Microsoft Excel (Microsoft Corp., Redmond, WA, USA). Normality was assessed using the Shapiro–Wilk test. Given the small cohort and sparse subgroups, nonparametric methods were preferred. Continuous variables are reported as medians with interquartile ranges (IQRs), with means and standard deviations (SDs) provided where appropriate. Categorical variables are presented as frequencies and percentages.

Continuous variables were compared between two independent groups using the Mann–Whitney U test. Categorical variables were analyzed using Fisher’s exact test. Associations between continuous variables, including body mass index (BMI) Z-scores and ppFEV_1_, were assessed using Spearman’s rank correlation coefficient (*ρ*). All tests were two-tailed, with statistical significance set at *p* < 0.05. No adjustment for multiple comparisons was applied. Primary between-group analyses were unadjusted and were interpreted as exploratory descriptive associations.

For analyses by CFTR modulator therapy status, ETI-treated patients (*n* = 35) were compared with a non-ETI group comprising LI-treated (*n* = 4) and modulator-naïve patients (*n* = 7). Continuous and categorical variables were compared using the Mann–Whitney U test and Fisher’s exact test, respectively. Comparisons were considered exploratory because of the marked age imbalance between groups and the small subgroup sizes.

To account for this age imbalance, secondary age-adjusted analyses were performed for the main clinical outcomes. BMI Z-score and ppFEV_1_ were analyzed using linear regression models including treatment group and age at follow-up as predictors. Inpatient days were analyzed using negative binomial regression with treatment group and age as predictors because of the count nature and skewed distribution of this outcome. Adjusted analyses were not performed for microbiological outcomes because of sparse event counts. These secondary analyses were considered exploratory.

A secondary, post hoc analysis was performed among the 10 patients recorded as Bihor County residents. The birth window was divided into pre-screening (2008–June 2022) and post-screening (July 2022–2024) periods. Based on approximately 4600 annual live births [22], cumulative denominators were estimated as 66,700 (14.5 years) and 11,500 (2.5 years) live births, respectively. Case-to-live-birth ratios were calculated with exact Poisson 95% confidence intervals. These estimates are not interpreted as population-based incidence rates.

Missing data were minimal across the dataset. Spirometric data were unavailable for 8 patients (17.4%) who were below the age threshold for reliable testing. No imputation methods were applied; analyses were performed on available cases.

### 2.4. Ethical Considerations

This study was conducted in accordance with the Declaration of Helsinki. Ethical approval was obtained from the Institutional Ethics subcommittee of University of Oradea on 30 January 2026, under registration number 1206/30.01.2026 and approval number 66. To ensure patient privacy, all data were fully anonymized prior to analysis, and no identifiable personal information was included in the research dataset.

### 2.5. Disclosure of Generative AI Use

Generative AI (ChatGPT Model 5.5) was utilized during the preparation of this manuscript for the purpose of technical text editing, structural organization, and translation assistance to ensure academic linguistic standards. AI was not involved in data collection, primary analysis, or the interpretation of clinical results.

## 3. Results

### 3.1. Demographic Characteristics and Regional Case-Detection Ratios in Bihor County

The study cohort comprised 46 patients diagnosed with CF across western Romania (the complete, fully anonymized regional dataset is available in Supplementary Table S1). Within this cohort, 27 patients are male (58.70%) and 19 are female (41.30%). The median age at follow-up (2025) was 8.96 years (IQR 7.06 to 15.25; range 1.20 to 19.63 years). The median BMI Z-score was −0.32 (IQR, −0.89 to 0.43; *n* = 46). Valid spirometry was available for 38 patients, with a median ppFEV_1_ of 99.0% (IQR, 92.25–107.5).

Ten patients (21.7%) were residents of Bihor County, corresponding to 1.82 currently followed cases per 100,000 inhabitants, based on an estimated population of 550,000. Using approximately 4600 annual live births [22], the overall case-to-live-birth ratio for 2008–2024 was 1:7820 (0.128 per 1000; 95% CI, 0.061–0.235). The corresponding ratios were 1:8338 before NBS implementation (eight cases; January 2008–June 2022) and 1:5750 afterward (two cases; July 2022–December 2024), representing a descriptive 1.45-fold increase in the post-screening case-detection ratio. Of the two post-screening-period patients, one was detected through NBS and one presented with MI. These estimates represent case-detection ratios rather than population-based prevalence or incidence.

### 3.2. Diagnostic Pathway and Age at Diagnosis

Five patients (10.9%) were screen-detected: four through the national NBS program after its implementation in July 2022 and one through privately funded screening in 2017. The remaining 41 patients (89.1%) were symptom-detected, including five who presented with MI at birth. Two of these five were born during the post-screening period but were classified as symptom-detected because diagnostic evaluation was initiated by their clinical presentation. Among the other 36 symptom-detected patients, evaluation was prompted primarily by recurrent respiratory infections and/or malnutrition associated with pancreatic insufficiency.

Age at diagnosis was significantly lower in screen-detected patients (median, 0.00 years; IQR, 0.00–0.20) than in symptom-detected patients (median, 0.60 years; IQR, 0.20–2.00; *p* = 0.016). Two symptom-detected patients were diagnosed at 13 and 17 years of age. Patients presenting with MI were also diagnosed earlier than those without this presentation (median, 0.00 vs. 0.60 years; *p* = 0.010). Age at diagnosis was not significantly associated with current BMI Z-score (*ρ* = −0.059, *p* = 0.696), ppFEV_1_ (*ρ* = −0.033, *p* = 0.842), or hospitalization days during 2025 (*ρ* = −0.176, *p* = 0.243).

### 3.3. Genetic Profile and Genotype-Phenotype Correlations

The F508del variant was identified in 60 of 92 alleles (65.2%). Genotype distribution by F508del status is shown in Table 1.

Among the remaining 32 non-*F508del* alleles distributed across the heterozygous and complex non-*F508del* subgroups, the specific molecular variants identified included G542X (*n* = 4 alleles), CFTRdele2,3 (*n* = 3 alleles), E831X (*n* = 3 alleles), alongside two alleles each for C524X, G85E, 1677delTA, 2184insA, G126D, and N1303K. Ten additional variants were identified once each: 1898+1G->A, E379X, C276X, c.1853_1863del, 3272-26A->G, R1066C, G576A, W1282X, D1152H, and CFTRdele16-20.

Valid spirometry was available for 38 patients. Median ppFEV_1_ was 99% (IQR, 91–108) in F508del-homozygous patients (*n* = 12) and 98% (IQR, 92–106) in those with other genotypes (*n* = 26), with no significant difference between groups (*p* = 0.671). Median BMI Z-score was also similar between F508del-homozygous patients and those with other genotypes (−0.78 [IQR, −1.30 to 0.38] vs. −0.31 [IQR, −0.86 to 0.43]; *p* = 0.641).

### 3.4. Clinical Characteristics According to CFTR Modulator Regimen

At evaluation, 35 patients (76.1%) were receiving ETI, 4 (8.7%) LI, and 7 (15.2%) were modulator-naïve. For analytic purposes, LI-treated and modulator-naïve patients were combined into a non-ETI comparator (*n* = 11). Clinical and microbiological characteristics by modulator status are summarized in Table 2.

The ETI group was substantially older than the non-ETI group (Table 2), reflecting age-based eligibility and the timing of reimbursement. BMI Z-score did not differ significantly between ETI and non-ETI patients (−0.22 vs. −0.45; *p* = 0.403). Median hospitalization burden during 2025 was numerically lower in ETI-treated patients (5.0 vs. 10.0 days; *p* = 0.069), and nine ETI-treated patients (25.7%) had no admissions. PsA colonization was less frequent in the ETI group (5.7% vs. 27.3%), though the difference did not reach significance (*p* = 0.080). MSSA and MRSA distributions did not differ between groups.

Among the 38 patients with valid spirometry (34 ETI, 4 non-ETI), median ppFEV_1_ predicted was comparable between groups (98.5% vs. 99.5%; *p* = 0.849); however, given the small non-ETI subgroup (*n* = 4), this comparison should be interpreted cautiously. Across the full spirometry subgroup, ppFEV_1_ was positively correlated with BMI Z-score (*ρ* = 0.625, *p* < 0.001) and inversely correlated with age at follow-up (*ρ* = −0.355, *p* = 0.029). Patients colonized with PsA had lower ppFEV_1_ (*p* = 0.021).

In secondary age-adjusted analyses, no significant between-group differences were identified. The adjusted mean difference for BMI Z-score was 0.53 (95% CI, −0.40 to 1.45; *p* = 0.256), and that for ppFEV_1_ was 7.76 percentage points (95% CI, −13.02 to 28.53; *p* = 0.453). In negative binomial regression, ETI treatment was not significantly associated with inpatient-day rate after adjustment for age (IRR = 0.78, 95% CI, 0.28–2.17; *p* = 0.630).

Cumulative inpatient days were inversely correlated with age at follow-up (*ρ* = −0.377, *p* = 0.010) and were higher in patients colonized with at least one pathogen (median 8 vs. 3 days; *p* = 0.018). Screen-detected patients had more cumulative inpatient days than symptom-detected patients (median 11 vs. 5 days; *p* = 0.019); however, this finding should be interpreted cautiously given the small screen-detected subgroup (*n* = 5), its predominantly young age distribution, and the influence of one patient who accumulated 66 inpatient days. MSSA-positive patients accumulated more inpatient days than MSSA-negative patients (median 15 vs. 5; *p* = 0.009), although MSSA positivity was not associated with ppFEV_1_ (*p* = 0.535) or BMI Z-score (*p* = 1.000). Pathogen-positive patients were younger at follow-up (*p* = 0.035); unstandardized anthropometric differences (weight, *p* = 0.019; raw BMI, *p* = 0.020) disappeared after age-standardization (BMI Z-score, *p* = 0.185).

Among ETI-treated patients, median exposure duration was approximately 2.0 years (IQR, 1.5–3.0), consistent with treatment initiation from approximately mid-2022 onward. Exposure duration was not significantly associated with hospitalization burden (*p* = 0.222). Hospitalization days were not significantly associated with BMI Z-score (*p* = 0.463) or ppFEV_1_ (*p* = 0.968). ppFEV_1_ was not significantly associated with hospitalization days, age at diagnosis, genotype, sex, or county of residence.

## 4. Discussion

To our knowledge, this is the first report that provides a baseline characterization of a regional CF cohort from western Romania during a period of concurrent transitions in NBS and CFTR modulator access.

### 4.1. Age at Diagnosis: Screen-Detected Versus Symptom-Detected Patients

Screen-detected patients were diagnosed significantly earlier than symptom-detected patients (median 0.00 vs. 0.60 years; *p* = 0.016), and later year of birth was associated with younger age at diagnosis (*ρ* = −0.577, *p* < 0.001). These findings align with the well-established benefits of NBS: in the United States, the post-NBS cohort had a median age at diagnosis of 0 months compared with 4 months in the pre-NBS era, with 92% of screened infants diagnosed within the first 6 months of life [23]. The Wisconsin randomized trial and subsequent registry analyses have confirmed that neonatal screening enables timely treatment initiation and is associated with improved nutritional and, in many studies, pulmonary outcomes [10]. The European Cystic Fibrosis Society Best Practice Guidelines recommend that screen-positive infants be evaluated by a CF specialist team by 35 days and no later than 58 days of life, positioning NBS as a cornerstone of early, potentially disease-modifying intervention in the era of HEMT [24].

However, the present cohort illustrates the limitations of a recently implemented program. Only five patients (10.9%) were screen-detected, as the national NBS program became operational only in July 2022 and the majority of the cohort was born before that date. Two post-screening-period patients were still diagnosed symptomatically because MI prompted clinical evaluation before NBS results became available, a recognized limitation [25]. Two additional symptom-detected patients were not diagnosed until ages 13 and 17 years, underscoring the risk of delayed recognition in pancreatic-sufficient or monosymptomatic presentations [26]. Delayed diagnosis remains a major barrier in lower-income settings, where median ages at diagnosis of 1.5–2.8 years have been reported and earlier PsA acquisition has been observed compared with North American registry populations [13,27].

Despite earlier diagnosis in screen-detected patients, age at diagnosis was not significantly associated with current BMI Z-score or hospitalization days. This likely reflects the small number of screen-detected patients, and the short follow-up since NBS implementation.

### 4.2. Clinical Characteristics According to CFTR Modulator Status

At the time of evaluation, 84.8% of the cohort was receiving CFTR modulator therapy (76.1% ETI, 8.7% LI), reflecting rapid uptake following the introduction of national therapeutic protocols in 2022 [18,19].

Median ppFEV_1_ was comparable between ETI-treated and non-ETI patients (98.5% vs. 99.5%; *p* = 0.849). After adjustment for age, the estimated between-group difference was 7.76 percentage points but remained non-significant and imprecise (95% CI, −13.02 to 28.53; *p* = 0.453), reflecting in part the availability of spirometry in only 4 of 11 non-ETI patients. The overall median ppFEV_1_ of 99.0% is broadly comparable to ECFSPR 2024 data for children aged 6–11 (101.7%) and 12–17 years (96.8%), as well as UK values (Table 3) [14,28]. These findings are consistent with the durable lung function improvements demonstrated in the PROMISE study, the German CF Registry, and the 192-week ETI open-label extension [29,30,31].

The strong positive correlation between ppFEV_1_ and BMI Z-score (*ρ* = 0.625, *p* < 0.001) is consistent with Grade I evidence from the Academy of Nutrition and Dietetics that maintaining BMI above the 50th percentile from early childhood is associated with better long-term ppFEV_1_, and with meta-analytic data showing significantly higher ppFEV_1_ in normal-weight versus underweight patients [32,33]. The inverse correlation with age (*ρ* = −0.355, *p* = 0.029) reflects the expected age-related decline in lung function, though this trajectory appears attenuated in the modulator era, with ETI-treated patients showing no pulmonary function loss over 2 years compared with a 1.92 percentage-point annual decline in untreated controls [34].

Overall cohort nutritional status remained below Western European benchmarks, with a median BMI Z-score of −0.32 compared with the ECFSPR 2024 median for children aged 2–17 (−0.1) and the UK value (0.2) (Table 3). Although the difference between ETI and non-ETI groups was not significant (−0.22 vs. −0.45; *p* = 0.403), it remained non-significant after adjustment for age (adjusted mean difference, 0.53; 95% CI, −0.40 to 1.45; *p* = 0.256). The direction is consistent with nutritional improvements reported in clinical trials and real-world registries: the pivotal ETI trial involving patients carrying one F508del allele and one minimal function CFTR variant demonstrated a BMI Z-score increase of 0.30 (95% CI, 0.17–0.43) at 24 weeks, the Danish CF Registry showed a mean BMI Z-score increase from −0.22 to 0.24 over 2 years of ETI in children, and the Italian National Registry reported a BMI Z-score increase of 0.2 SD in children over 24 months [35,36,37]. The suboptimal nutritional status may reflect the legacy of delayed diagnosis in the pre-screening era, socioeconomic factors, or differences in nutritional support infrastructure—consistent with ECFSPR data demonstrating that patients in lower-income European countries have significantly higher rates of underweight status and that avoiding underweight would increase survival by up to 42 years in these populations [38].

Median hospitalization days were numerically lower in ETI-treated patients (5.0 vs. 10.0; *p* = 0.069), consistent with the substantial reductions documented in pivotal trials (71% reduction in odds of hospitalization) and US registry data (65% reduction in pediatric hospitalizations between 2018 and 2022) [39,40]. After adjustment for age, ETI treatment was not significantly associated with inpatient-day rate (IRR = 0.78, 95% CI, 0.28–2.17; *p* = 0.630). The younger age of the non-ETI group remains relevant when interpreting these findings, particularly because it included infants requiring more intensive monitoring. The counterintuitive finding that screen-detected patients had more inpatient days (median 11 vs. 5; *p* = 0.019) reflects the composition of this subgroup—predominantly very young patients in the intensive initial management phase, with one patient accumulating 66 days.

Chronic PsA colonization was less frequent in the ETI group (5.7% vs. 27.3%; *p* = 0.080). The pediatric rate of 11.4% exceeds the ECFSPR pediatric rate (7.4%) and the UK pediatric rate (2.5%) (Table 3). The lower rate in ETI-treated patients is consistent with evidence that CFTR modulators improve mucociliary clearance and reduce pathogen density, although most patients with established chronic infection remain persistently infected [41,42]. The MRSA chronic colonization rate (15.2% overall) exceeds UK prevalence (~3.4%) and is consistent with the known regional variability in MRSA prevalence across CF centers (3–26%) [43]. MSSA-positive patients accumulated more inpatient days (median 15 vs. 5; *p* = 0.009) without associated differences in ppFEV_1_ or BMI Z-score, suggesting the hospitalization burden may reflect treatment intensity rather than direct pulmonary impact. Turkish CF Registry data demonstrated that modulator therapy significantly reduced chronic colonization rates for PsA, MSSA, and MRSA, with 44% of PsA-colonized children achieving culture-negative status [44].

Taken together, these pulmonary, nutritional, hospitalization, and microbiological findings should be interpreted within the broader European context summarized in Table 3. Although differences in cohort composition, denominators, and reporting methods preclude direct equivalence, CFTR modulator uptake (84.8%) was the same reported percentage as in the UK Registry, while median ppFEV_1_ (99.0%) fell within the pediatric ECFSPR IQRs. Conversely, median BMI Z-score was lower than the ECFSPR and UK pediatric medians (−0.32 versus −0.1 and 0.2), while chronic PsA was more frequent (11.4% versus 7.4% and 2.5%). These comparisons should be interpreted cautiously because differences in patient characteristics, diagnostic history, surveillance practices, and outcome definitions limit direct comparability between cohorts.

Within this evolving care landscape, the near-contemporaneous introduction of universal NBS and ETI reimbursement in 2022 may facilitate earlier specialist care and disease-modifying treatment [26,45]. ECFSPR-based modelling projected that modulator access could increase survival by 15–29 years in lower-income European settings [38], while the ECFS/Cystic Fibrosis Europe Twinning Project provides a structured framework for harmonizing care [46]. Comprehensive national registry coverage, sustained NBS quality assurance, strengthened multidisciplinary care, and longitudinal follow-up remain priorities for determining whether these single-center findings translate into durable population-level benefits.

### 4.3. Regional Case-Detection Ratios in Bihor County

The third aim was to estimate regional case-detection ratios in Bihor County across pre-screening and post-screening birth periods. The overall case-to-live-birth ratio for 2008–2024 was 1:7820 (95% CI, 0.061–0.235 per 1000), with a descriptive 1.45-fold higher detection ratio in the post-screening period (1:5750) compared with the pre-screening period (1:8338). These estimates represent case-detection ratios rather than population-based incidence rates, given the small number of cases (*n* = 10), the single-center design, and the short post-screening observation window.

The pre-screening ratio of 1:8338 is lower than the commonly cited European CF incidence of approximately 1:3000–6000 and lower than reported figures for neighboring Eastern European countries such as the Czech Republic (1:2833), Slovakia (1:1800), and Bulgaria (1:2500) [26,47]. This discrepancy likely reflects underdiagnosis rather than a genuinely lower disease frequency. Romania was among the non-EU countries with the lowest CF prevalence estimates in the European Cystic Fibrosis Demographics Registry, and the median age in non-EU countries was nearly 5 years younger than in EU countries, suggesting both underdiagnosis and premature mortality [47]. Globally, an estimated 76,569 people with CF remain undiagnosed across 55 countries, with 82% living in low- and middle-income countries [48]. Even in high-income settings, ethnic minority populations may be systematically underdiagnosed due to NBS panels designed around common Northern European variants [13].

The descriptive increase in the case-detection ratio following NBS implementation is consistent with the expected effect of systematic screening, although the small numbers (2 post-screening cases, one detected by meconium ileus rather than NBS) preclude definitive conclusions. Continued surveillance over a longer post-screening period will be essential to determine whether the case-detection ratio approaches the expected European incidence range and to quantify the true burden of CF in this region.

### 4.4. Genetic Profile

Although not a primary aim, the genetic profile of this cohort merits discussion. The F508del variant accounted for 65.2% of alleles; 37.0% of patients were homozygous and 56.5% compound heterozygous. This frequency is higher than that reported in Poland (54.5%) but lower than in the United States (~85%) and Denmark (87.2%), consistent with the northwest-to-southeast European gradient in F508del prevalence [49,50].

Non-F508del variants reflected the genetic heterogeneity of Southeastern Europe, including G542X and 1677delTA [50]. Notably, the Slavic-origin CFTRdele2,3 variant was identified in three alleles, adding to the documented genetic diversity of Romanian CF populations [51].

The absence of significant differences in ppFEV_1_ or BMI Z-score between F508del-homozygous patients and other genotypes may reflect the high uptake of CFTR modulators and the predominantly pediatric cohort, whose preserved lung function may limit detectable genotype–phenotype differences [45].

### 4.5. Limitations

Several limitations should be acknowledged. First, the small sample size (*n* = 46) limits statistical power and the number of covariates that could be included in multivariable models; therefore, the age-adjusted analyses were restricted to treatment group and age and should be considered exploratory. Second, the cross-sectional design cannot establish causal relationships between modulator therapy and clinical outcomes, and residual confounding may persist despite adjustment for age. Third, spirometric data were unavailable for 8 patients (17.4%) below the age threshold for reliable testing, and only 4 non-ETI patients had valid spirometry, limiting the interpretability of lung function comparisons. Fourth, the single-center design may not be representative of the national CF population, and the Bihor County epidemiological estimates are based on very small numbers with wide confidence intervals. Fifth, the comparisons with ECFSPR and UK registry data in Table 3 are contextual only; despite age stratification where applicable, differences in case mix, calendar year, culture frequency, and endpoint definitions limit direct comparability, and no statistical comparison or equivalence claim is intended. Finally, the short duration since NBS implementation (~3 years) and modulator availability (~3 years) limits the ability to assess long-term outcomes. Multi-center studies with larger, nationally representative cohorts are needed to confirm these findings.

## 5. Conclusions

This study provides an initial characterization of a regional CF cohort in western Romania during a transformative period in screening and treatment. NBS was associated with substantially earlier diagnosis, while CFTR modulator uptake was rapid and comparable to Western European benchmarks. Lung function in the spirometry-eligible subgroup was broadly comparable to European registry values. Nevertheless, persistent nutritional deficits, higher chronic PsA and MRSA colonization rates than in Western European populations, and low case-detection ratios suggesting continued underdiagnosis highlight important gaps in care. Sustained NBS implementation, improved diagnostic awareness, and comprehensive nutritional and microbiological management will be essential to strengthen CF care in Romania. Longitudinal follow-up integrated with national registry data will be needed to determine whether recent advances translate into sustained improvements in clinical outcomes.

## Figures and Tables

**Table 1:** Distribution of CFTR genotype status within the regional cystic fibrosis cohort.

Genotype Status	Frequency (*n*)	Percentage (%)
Homozygous *F508del* (Δ*F508* Δ*F508*)	17	36.96%
Heterozygous *F508del* (Δ*F508* Other Mutation)	26	56.52%
Other CFTR Mutations (Non-*F508del*)	3	6.52%
Total	46	100.0%

**Table 2:** Clinical characteristics at the 2025 follow-up according to current CFTR modulator regimen.

Parameter	ETI (*n* = 35)	Non-ETI ^†^ (*n* = 11)	Unadjusted *p*	Age-Adjusted Estimate (95% CI)
Age at Follow-up, years	11.46 (8.25–16.09)	2.96 (1.71–7.04)	<0.001	−
BMI Z-score	−0.22 (−0.82 to 0.56)	−0.45 (−1.16 to 0.01)	0.403	β * = 0.53 (95% CI −0.40 to 1.45); *p* = 0.256
ppFEV1%	98.50 (92.25–107.50) [*n* = 34]	99.50 (92.00–102.25) [*n* = 4]	0.849	β = 7.76 (95% CI −13.02 to 28.53); *p* = 0.453
Inpatient days during 2025	5.00 (0.50–8.00)	10.00 (3.50–20.00)	0.069	IRR = 0.78 (95% CI 0.28–2.17); *p* = 0.63
Chronic PsA Colonization (*n*, %)	2 (5.71%)	3 (27.30%)	0.080	−
Chronic MSSA Colonization (n, %)	7 (20.00%)	0 (0.00%)	0.171	−
Chronic MRSA Colonization (n, %)	6 (17.14%)	1 (9.10%)	1.000	−

^†^ Non-ETI group comprises 4 patients receiving LI and 7 modulator-naïve patients. * β denotes the adjusted mean difference. Continuous variables are presented as median (IQR). Unadjusted comparisons used the Mann–Whitney U test for continuous variables and two-sided Fisher’s exact test for categorical variables. Age-adjusted analyses used linear regression for BMI Z-score and ppFEV_1_ and negative binomial regression for inpatient days. Adjusted analyses were not performed for colonization outcomes because of sparse event counts. Spirometry was available for 34 ETI and 4 non-ETI patients, all of whom were modulator-naïve. CI, confidence interval; ETI, elexacaftor/tezacaftor/ivacaftor; IRR, incidence rate ratio; IQR, interquartile range; LI, lumacaftor/ivacaftor; ppFEV_1_, percent-predicted forced expiratory volume in 1 s; PsA, *Pseudomonas aeruginosa*; MSSA, methicillin-sensitive *Staphylococcus aureus*; MRSA, methicillin-resistant *Staphylococcus aureus*.

**Table 3:** Descriptive comparison between the Western Romania cohort and the 2024 ECFSPR, including UK data.

Indicator	Western Romania Cohort	ECFS Patient Registry	UK Cystic Fibrosis Registry
Median age at follow-up, years	8.96 (IQR 7.06–15.25)	20.8	23.6
CFTR modulator therapy	39/46 (84.8%)	-	8830/10,414 (84.8%)
ppFEV_1_, median (IQR)	6–11 yr: 102.5 (96.0–117.3), *n* = 20 12–17 yr: 95.5 (90.3–103.5), *n* = 16	6–11 yr: 101.7 (91.5–111.2) 12–17 yr: 96.8 (85.6–106.4)	6–11 yr: 101.8 (93.7–110.3) 12–17 yr: 97.1 (88.4–105.6)
BMI Z-score, median (IQR)	2–17 yr: −0.27 (−0.94 to 0.44), *n* = 40	2–17 yr: −0.1 (−0.8 to 0.6)	2–17 yr: 0.2 (−0.4 to 0.9)
Chronic PsA colonization	Children: 5/44 (11.4%) Adults: 0/2 (0.0%)	Children: 7.4% Adults: 26.2%	Children: 2.5% Adults: 11.6%

UK comparator data are from ECFSPR 2024, except CFTR modulator use, which is from the UK CF Registry 2024. Western Romania data were age-stratified where applicable. Comparisons are descriptive because cohort characteristics and definitions differ; the western Romania adult subgroup included only two patients.

## Data Availability

The original contributions presented in this study are included in this article/Supplementary Materials. Further inquiries can be directed to the corresponding author.

## Supplementary Materials

The following supporting information can be downloaded at: https://www.mdpi.com/article/10.3390/children13091178/s1, Supplementary Table S1: Complete anonymized demographic, genetic, and clinical dataset of the regional western Romania cystic fibrosis cohort.

## Abbreviations

The following abbreviations are used in this manuscript:

BMI	Body Mass Index
cAMP	Cyclic Adenosine Monophosphate
CF	Cystic Fibrosis
CFTR	Cystic Fibrosis Transmembrane Conductance Regulator
ECFSPR	European Cystic Fibrosis Society Patient Registry
ETI	Elexacaftor/Tezacaftor/Ivacaftor
FEV_1_	Forced Expiratory Volume in 1 Second
HEMT	Highly Effective Modulator Therapy
IQR	Interquartile Range
LI	Lumacaftor/Ivacaftor
MI	Meconium ileus
MRSA	Methicillin-Resistant Staphylococcus aureus
MSSA	Methicillin-Susceptible Staphylococcus aureus
NBS	Newborn Screening
PsA	Pseudomonas aeruginosa
ppFEV_1_	percent-predicted Forced Expiratory Volume in 1 Second
SD	Standard deviation
